# How shall we treat locally advanced triple negative breast cancer?

**DOI:** 10.12688/f1000research.20509.2

**Published:** 2020-05-04

**Authors:** Paulo Luz, David Dias, Ana Fortuna, Luis Bretes, Beatriz Gosalbez

**Affiliations:** 1 Medical Oncology, Centro Hospitalar Universitário do Algarve, Faro, Portugal

**Keywords:** triple negative, breast cancer, neoadjuvant chemotherapy

## Abstract

Triple negative breast cancer (TNBC) has been shown to respond to neoadjuvant chemotherapy (NACT). It has been established that achieving pathological complete response (pCR) for certain aggressive subtypes of breast cancer, including HER-2 (over-expressed) and TNBC, provides an important surrogate marker for predicting long-term clinical response and survival outcomes.

How to increase the number of patients that achieve pCR remains challenging. Platinum-based NACT seems to be part of the solution and capecitabine, an active drug in metastatic breast cancer, but not a standard one in earlier stages may have found its place in the adjuvant setting. In the near future immunotherapy can play a role in early TNBC

Triple negative breast cancer (TNBC) is immunohistochemically defined as the lack of expression of estrogen, progesterone receptor and human epidermal growth factor receptor 2 (HER-2). It accounts for 15–20% of breast cancer cases and is characterized as a molecular heterogeneous disease that usually presents an aggressive clinical behavior and higher prevalence in younger women ^1^. Once TNBC has metastasized, it has the worst prognosis and the shortest OS of all breast cancer subtypes. On the other hand, TNBCs are highly chemo-sensitive and have been shown to respond very well to neoadjuvant chemotherapy (NACT) ^1– 3^.

The main goal of NACT strategies are not only to decrease the need for radical mastectomy but also to obtain an important predictive marker of favorable prognosis - the pathological complete response (pCR) - which is defined as the absence of invasive tumor cells (ypT0/is, ypN0/is). A correlation was observed between the pCR and the overall survival (OS) and disease free survival (DFS) outcomes in all subtypes of breast cancer, especially in aggressive ones such as HER2 positive and TNBC ^2^. The pCR achieved by NACT represents to date the ideal endpoint in translational investigation of biomarkers and pharmacological treatments. Patients who do not have pCR after NACT with the combination of taxane and anthracycline have 20 to 30% risk of relapse ^2, 3^.

How to increase the number of patients that achieve pCR remains challenging. Platinum-based NACT seems to be part of the solution. A metanalysis that enrolled nine randomized clinical trials (RCT) with 2109 patients showed that platinum-based NACT compared to platinum-free NACT significantly increased pCR rate from 37.0% to 52.1% ^4^. However, only two RCTs (Gepar-Sixto trial and CALGB 40603) reported survival outcomes: no significant difference in event free survival (EFS) and OS was observed in the combined analysis ^5, 6^. In the CALGB study ^6^ adding carboplatin did not significantly impact survival. The absolute benefit in 3-year event-free survival of adding carboplatin was 4.9% and the OS differences were also not significant, with 81,9% OS in the carboplatin group versus 84,6% without carboplatin. These results conflict with those in the Gepar-Sixto trial ^5^. In the TNBC subgroup, carboplatin resulted in a significantly improved pCR rate over control (53% vs 37%; P = 0.005). This translated into an absolute benefit in 3-year event EFS for the addition of carboplatin over control of 9.7% (85.8% vs 76.1%, respectively). What reasons can explain this discrepancy? There are several differences between these two studies worth noting. GeparSixto had more-favorable baseline characteristics, as 56% of patients were cN0, compared with 42% in CALGB, 76% of patients had tumors of high grade in CALGB, while 65% of tumors were high grade in GeparSixto, albeit in both TNBC and HER2. Long term survival analysis is needed. Additionally, a larger proportion were cT1 in GeparSixto (26% vs 11%). In CALGB 40603, the backbone therapy also included cyclophosphamide, which can also cause DNA damage like platinum agents, potentially making the treatment effect similar in the control and experimental arms ^5, 6^. Notably, BRCA-mutated patients experienced overall higher pCR rates, although no benefit was observed with the incorporation of platinum agents ^4^.

Current controversy in this field also includes the benefit of additional therapy after surgery. Given the still significantly high rates of residual disease after neoadjuvant therapy in TNBC, which portends inferior DFS, another approach to improving outcomes in this population is to add additional adjuvant therapy after surgery. The great majority of the studies focus on capecitabine, an active drug in metastatic TNBC. In the CREATE X trial (Capecitabine for Residual Cancer as Adjuvant Therapy) ^7^ patients who have not achieved pathologic complete response after undergoing neoadjuvant therapy were randomized to receive standard treatment either with capecitabine or without (control). Among patients with triple negative disease, the rate of disease-free survival was 69.8% in the capecitabine group versus 56.1% in the control group, and the overall survival rate was 78.8% versus 70.3% ^7^. At the San Antonio Breast Cancer Symposium 2018, the results of the phase III trial conducted by the Spanish group and the Central and South American group were presented, where treating patients with early-stage TNBC with capecitabine after surgery and standard chemotherapy did not significantly improve disease-free or overall survival compared with observation group ^8^. One possible explanation for the discrepancy between the results of the CREATE-X trial and this trial may be the different prognostic features between the populations. The risk of relapse of the population was much lower than in the CREATE-X trial. So, capecitabine, an active drug in metastatic breast cancer, but not a standard one in earlier stages may have found its place.

An important question remains, should standard of care with NACT on TNBC rely on platinum-based treatments with the intent of achieving higher pCR rates and a probable benefit in OS or should it continue with taxane and anthracycline based combinations and consider the use capecitabine when pCR is not feasible?

According to the authors´ perspectives, the impact of platinum on pCR and OS cannot simply be ignored. This option must be considered after balancing the potential benefits on disease outcomes versus increased toxicity. Special attention must be placed in older and frail patients that are still capable and willing to undergo NACT. In this subgroup of patients, taxane and anthracycline combination remains a valid first choice treatment and adjuvant capecitabine should be considered when residual tumor is still present. In young high risk patients who underwent platinum-based NACT and didn’t achieve pCR, adjuvant capecitabine should be discussed even we don’t have any RCT to support this idea.

Chemotherapy remains the backbone of TNBC, but research and development of new modalities of treatment continues. Recent and promising results were available in the metastatic setting with immunotherapy and in the near future that can play a role in early stage. KEYNOTE-522 ^9^ included 602 women with early TNBC and randomly assigned them to receive neoadjuvant therapy with four cycles of carboplatin/paclitaxel, followed by four cycles of anthracycline with or without pembrolizumab. After surgery, patients received nine cycles of adjuvant pembrolizumab or placebo. Both PD-L1–positive and PD-L1–negative patients achieved pCR. In PD-L1–positive patients, pCR were 68.9% vs 54.9%, respectively. In PD-L1–negative patients, pCR rates were 45.3% vs 30.3%, respectively. On the other hand, NeoTRIP ^10^ randomly assigned 280 women with early or locally advanced TNBC to receive neoadjuvant therapy with either atezolizumab plus carboplatin/nab-paclitaxel or placebo plus the same chemotherapy. All patients underwent surgery and then received four further cycles of anthracycline-based chemotherapy. pCR rates were not significantly different between the two study arms: 43.5% with atezolizumab vs 40.8% with chemotherapy alone. A multivariate analysis showed that the only variable associated with pCR rate was PD-L1–positive status ( *P* < .0001).

One of the possible explanations for the difference seen in these two trials may at least in part be related to the chemotherapy backbone. In the KEYNOTE-522 study, patients received anthracyclines in the neoadjuvant phase, whereas this was given after surgery without atezolizumab in NeoTRIP Moreover, there appeared to be patients with higher-risk disease in NeoTRIP compared with KEYNOTE-522: the first trial included patients with N3 disease (15% of patients), while the KEYNOTE-522 study excluded and more patients had stage IIIA and IIIB disease (49% vs. 25%).

More studies are needed to identify which patients will benefit and whether one checkpoint inhibitor is better than another. We hope that some of these drugs may soon have a role in the neoadjuvant setting. Right now, we know neoadjuvant treatment allows us to re-write the story right at the start and we just cannot miss that opportunity.

## Data availability

### Underlying data

No data are associated with this study.
