# Supplementary material for: How shall we treat locally advanced triple negative breast cancer?
Source: F1000Res. 2020 May 4;8:1649. Originally published 2019 Sep 16. [Version 2] doi: 10.12688/f1000research.20509.2 (PMC7411516; doi:10.12688/f1000research.20509.2)
Supplement: Peer review history

# Peer review: Reviewer response for version 2

**DOI:** 10.5256/f1000research.26216.r67500

**Published:** 2020-08-06

**Authors:** Bruna Cerbelli

**Affiliations:** 1 Medico-Surgical Sciences and Biotechnologies, Sapienza University, Latina, Rome, Italy

- The authors provide their opinion about the old and novel therapeutic strategy in non metastatic TNBC. 
- The trial is well analysed and commented; however, the manuscript requires some improvement of English.
- In the context of novel option, the authors should discuss the negative results of neoadiuvant GEpar nuevo study, presented at ASCO 2018 and the role of window study. 

Is the topic of the opinion article discussed accurately in the context of the current literature?

Yes

Are arguments sufficiently supported by evidence from the published literature?

Partly

Are all factual statements correct and adequately supported by citations?

Yes

Are the conclusions drawn balanced and justified on the basis of the presented arguments?

Yes

Reviewer Expertise:

NA

I confirm that I have read this submission and believe that I have an appropriate level of expertise to confirm that it is of an acceptable scientific standard.

# Peer review: Reviewer response for version 1

**DOI:** 10.5256/f1000research.22542.r58166

**Published:** 2020-01-21

**Authors:** Brian D. Lehmann

**Affiliations:** 1 Department of Medicine, Vanderbilt University Medical Center, Nashville, TN, USA

In this article the authors provide perspective on the use of platinum compounds in the treatment of early stage triple-negative breast cancer. The authors provide opinions on a recently published meta-analysis of nine randomized clinical trials (RCT) treated with platinum-based neoadjuvant chemotherapy.

 Specific comments:

1. Please finish the sentence, “Notably, BRCA-mutated patients experienced overall higher pCR rates, although no benefit was observed with the.” The sentence and idea trying to be presented is incomplete. 
2. The authors state, “What reasons can explain this discrepancy? There are several differences between these two-studies worth noting. GeparSixto had more-favorable baseline characteristics, as 26% of patients were cN0, compared with 42% in CALGB 40603.” This statement does not appear to be accurate. How is 26% of patients with cN0 in GeparSixto more favorable than 42% of patients with cN0 in CALGB? Please revise and double check the numbers. 42% is correct for CALGB as the patient population was limited to TNBC. GeparSixto included both TNBC and HER2 positive and at least in the combined population cN0 was 50%. Not sure what the value is when restricted to TNBC. The authors should consider comparing tumor grade, as higher grades are associated with better chemotherapy response. In CALGB 76% of patients had tumors of high grade, while 65% of tumors were high grade in GeparSixto, albeit in both TNBC and HER2. 
3. The statement, “Recently, at the San Antonio Breast Cancer Symposium 2018…)” is not so recent anymore. Consider removing “recent” or replacing with newer published data. 
4. The authors propose the very relevant question, “An important question remains, should standard of care with NACT on TNBC rely on platinum-based treatments with the intent of achieving higher pCR rates and a probable benefit in OS or should it continue with taxane and antracyclin based combinations and consider the use capecitabine when pCR is not feasible?” While the authors mention the data cannot be ignored, the authors do not mention how the question might finally be resolved. Especially given that there were no EFS or OS benefits from the two studies with published results, how can we justify the substantial toxicities of an additional platinum compound? Perhaps only when we see additional long-term analysis from the majority of RCTs with platinum compounds could the question be answered.
5. The authors conclude with, “We hope that some of these drugs, like immunotherapy, may soon have a role in the neoadjuvant setting.” While this may certainly be true in the metastatic setting, recent results suggest that this may not be the case in the neoadjuvant setting. Please include a discussion of immune checkpoint inhibitors in the neoadjuvant setting. Results from the NeoTRIPaPDL1 trial presented at SABC 2020 (Abstract #GS3-04) concluded that Atezolizumab with neoadjuvant chemotherapy does not improve pathologic complete response rates for patients with triple-negative breast cancer.

Is the topic of the opinion article discussed accurately in the context of the current literature?

Yes

Are arguments sufficiently supported by evidence from the published literature?

Partly

Are all factual statements correct and adequately supported by citations?

Yes

Are the conclusions drawn balanced and justified on the basis of the presented arguments?

Yes

Reviewer Expertise:

Triple-negative breast cancer, translational research and genomics

I confirm that I have read this submission and believe that I have an appropriate level of expertise to confirm that it is of an acceptable scientific standard.

# Response

**Published:** 2020-04-28

**Authors:** Paulo Luz

**Affiliations:** Centro Hospitalar Universitário do Algarve, Portugal

We appreciate the time and effort that you have dedicated to providing your valuable feedback on our manuscript. New evidence about immunotherapy in early TNBC is available (NeoTRIP and KEYNOTE 522). We incorporated that information in this new version of the article.

We have also been able to incorporate changes to reflect most of the suggestions provided by you.
